# Septate Uterus. Resect or not? That is Not the Only Question

**DOI:** 10.1055/s-0042-1754377

**Published:** 2022-08-29

**Authors:** Tayfun Cok

**Affiliations:** 1 Department of Obstetrics and Gynecology, School of Medicine, Adana Research and Education Centre, Baskent University, Adana, Turkey.

The septate uterus is the most common congenital anomaly of the uterus. The prevalence of septate uterus varies between 2.3 and 15.4% according to the population studied, with the percentages being higher in women with recurrent miscarriages combined with infertility. 1 A septate uterus increases adverse pregnancy outcomes such as miscarriage, preterm birth, and malpresentation of the fetus, although its direct association with infertility is likely but still controversial. 2 3 This issue has also provoked a vivid debate on hysteroscopic resection of the septate uterus for treatment. 4 Especially for the management of the partial uterine septum, the main point which affects the resection decision lies within the problem of diagnosis. There has been a continuous change in the diagnostic criteria of the partial septate uterus along with the new guidelines, since 1988, despite increasing diagnostic tools. 5 6 7 8 9 Even in the same group of patients, the prevalence of septate uterus has been reported to vary from 5 to 31%, according to the criteria that have been used. 10 One patient flying from Europe to the USA may lose her septum over the Atlantic Ocean. A patient with a 12 mm septal indentation, who used to have a septate uterus according to the American Fertility Society (1988), 5 lost her septum in 2016, according to the American Society for Reproductive Medicine (ASRM) 2016 7 guidelines and gained it back in the ASRM 2021 updated guidelines. 9 The European Society of Human Reproduction and Embryology (ESHRE) and the European Society for Gynaecological Endoscopy's (ESGE) shared guidelines (2016) 7 boosted the number of patients with a diagnosis of the septate uterus, whereas ASRM (2016) 7 underestimates the number of patients with partial septate uterus and leaves some patients in a gray zone. Some of this underestimation is due to the 90° angle criteria, which seem to be arbitrary and are not based on strong evidence. 10 In the only randomized controlled The Randomised Uterine Septum Trial (TRUST) of uterine septum resection, 90% of the patients had a partial septate uterus, and three different criteria were used throughout the study. However, most importantly, the difference in the diagnosis reflects in the preference of the treatment regarding resection, and the interpretation of the outcomes of septum resection according to various diagnostic criteria lacks guidance in clinical practice. 11 After the TRUST results, we have the option to discuss expectant management with patients. In those patients who choose expectant management, we can find out cut-off values for septal indentation length and the angle for poor obstetric outcomes, if there is any relation.
